# Supplementary material for: Rank and payoff biases influence subject choices in a foraging task among sanctuary chimpanzees (Pan troglodytes)
Source: Anim Cogn. 2026 Jun 26;29(1):65. doi: 10.1007/s10071-026-02079-9 (PMC13582275; doi:10.1007/s10071-026-02079-9)
Supplement: Supplementary file 1 [file 10071_2026_2079_MOESM1_ESM.pdf]

Supplementary material for: Rank and payoff biases influence subject choices in a foraging task among sanctuary chimpanzees (*Pan troglodytes*)

Supplementary Figure 1:

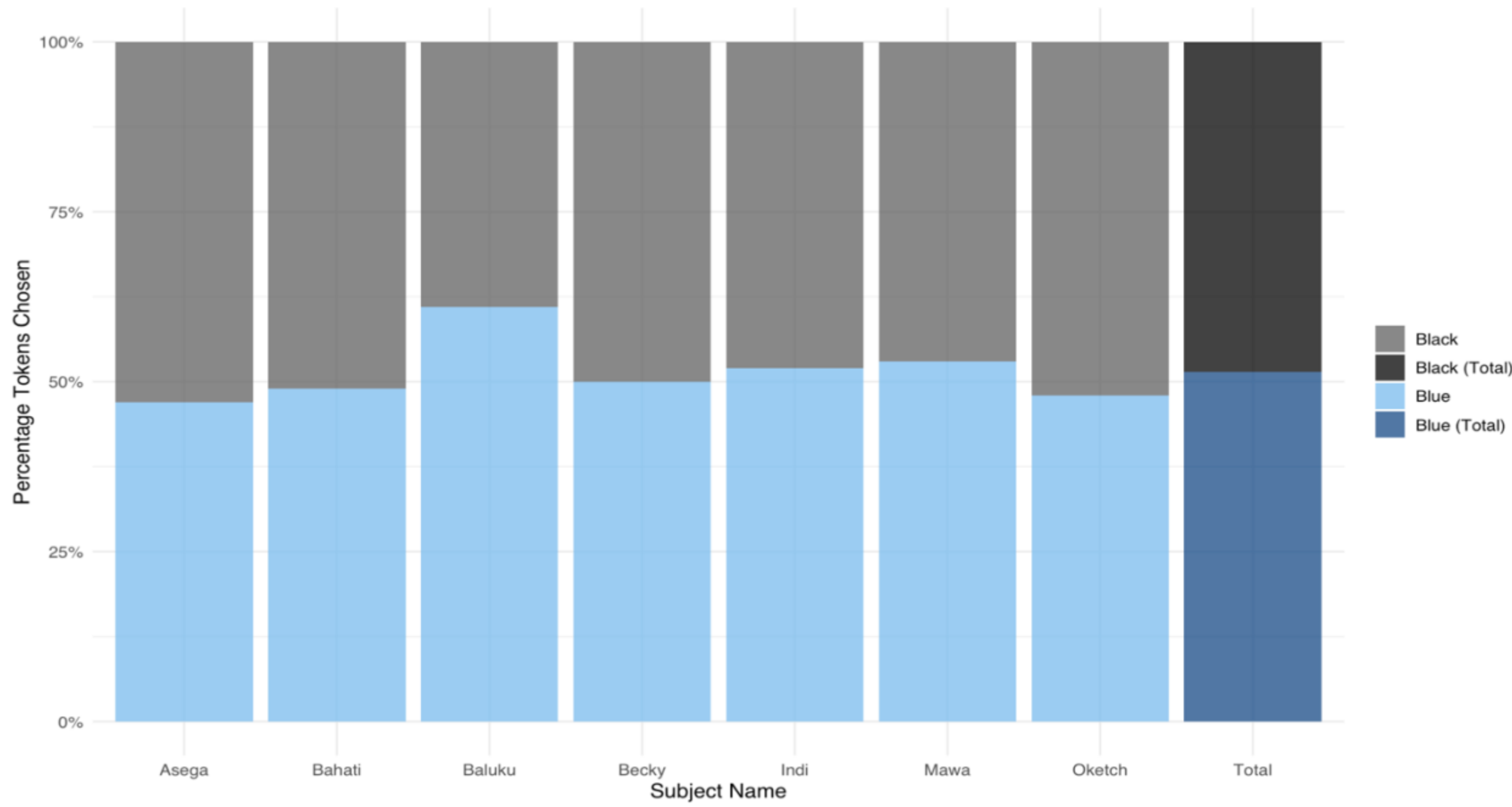

Supplementary Figure 2:

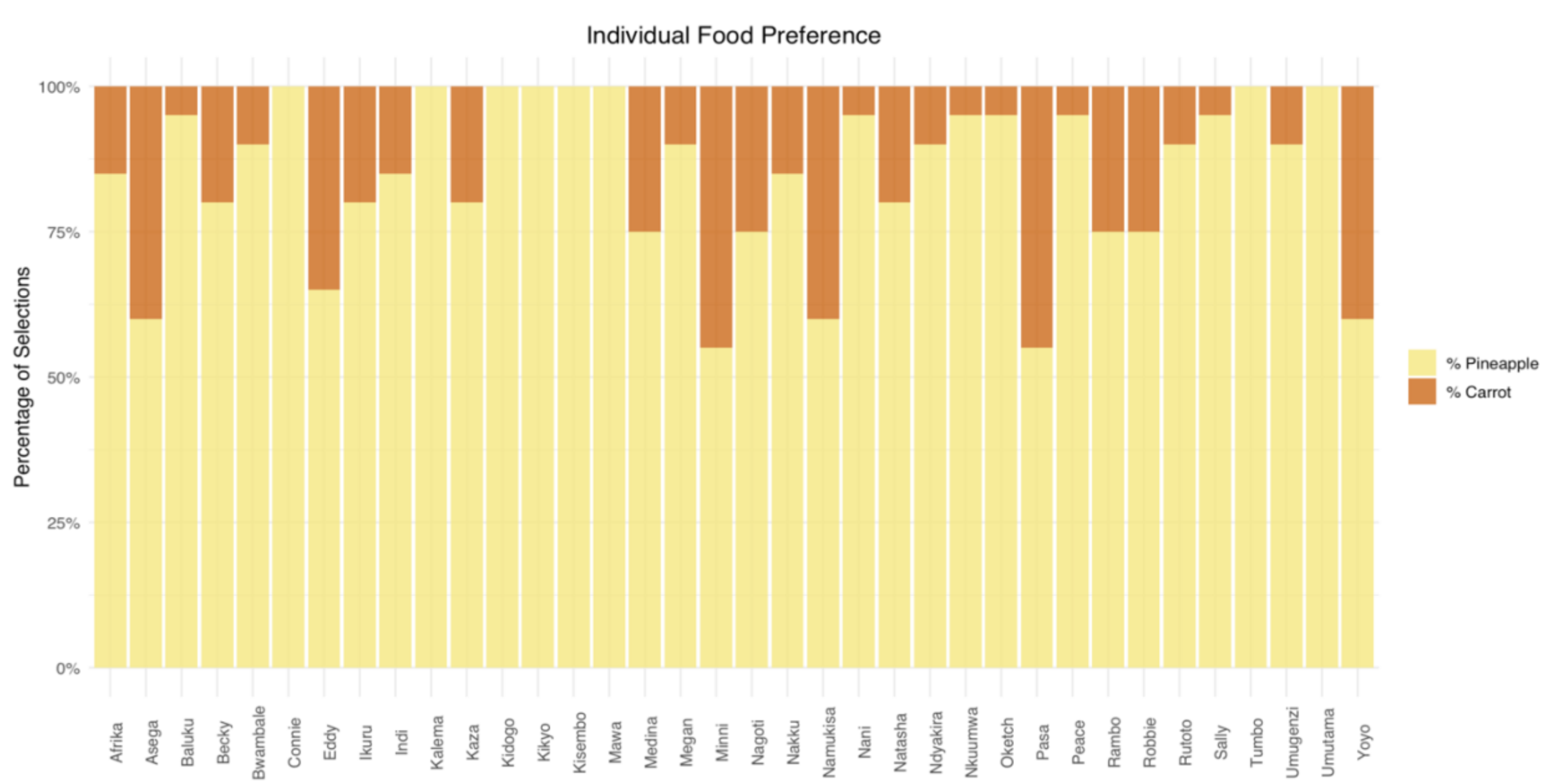

Supplementary Information 1:

Six individuals scored between 35% and 45% for carrot preference in the food preference test. One of these individuals was a demonstrator and another was in the control group. The four remaining participants (1 Group 1; 3 Group 2) were placed in a subset for further analysis to ensure food preference did not influence test results. Participants in this subset selected the HP token significantly more frequently than the LP token overall ($p<0.001$) and as their first choice ($p = 0.04$; Wilcoxon signed-rank), indicating their food preference score did not influence their performance in the experiment.
